# Prevalence of Carbapenem Non-susceptible Gram-Negative Bacteria at Tertiary Care Hospitals in Saudi Arabia

**DOI:** 10.7759/cureus.33767

**Published:** 2023-01-14

**Authors:** Rayan I Aloraifi, Abdullah F Alharthi, Abdulrahman A Almefleh, Abdulkhaleq H Alamri, Adi S Alobud, Reema A Bawazeer, Abdulrahman A Alswaji, Bassam Alalwan, Marwh G Aldriwesh, Sameera M Al Johani, Majed F Alghoribi

**Affiliations:** 1 College of Medicine, King Saud Bin Abdulaziz University for Health Sciences, Riyadh, SAU; 2 Infectious Disease Research, King Abdullah International Medical Research Center, Riyadh, SAU; 3 Microbiology Section, Pathology and Laboratory Medicine Department, King Abdulaziz Medical City, Riyadh, SAU; 4 Clinical Laboratory Sciences, College of Applied Medical Sciences, King Saud bin Abdulaziz University for Health Sciences, Riyadh, SAU

**Keywords:** surveillance, antimicrobial resistance, saudi arabia, antimicrobial resistance surveillance, gram-negative bacteria, carbapenem resistance

## Abstract

Background

Antibiotics significantly increased life expectancy and decreased mortality rates due to infections. However, this trend is starting to fade with the rise of multidrug-resistant organisms (MDR); these strains are becoming a global burden on healthcare and the economy. The dramatic increase and spread of carbapenem-resistant gram-negative bacteria (CRGNB) has become a serious global public health concern. In this retrospective cross-sectional study, we aimed to estimate the rates of gram-negative bacteremia in five tertiary care hospitals in different geographical locations in Saudi Arabia for five years.

Methods

A retrospective cross-sectional study was conducted in five tertiary care hospitals in Saudi Arabia among patients with bacteremia due to CRGNB. Electronic medical records were used to retrieve data regarding patient demographics and antimicrobial susceptibility testing (AST) over five years between January 2016 and December 2020. Patients with positive blood cultures for carbapenem-resistant *Escherichia (E.) coli, Klebsiella (K.) pneumonia, Pseudomonas (P.) aeruginosa, *and* Acinetobacter (A.) baumannii* comprise the final study population.

Results

This retrospective multicentric study was conducted between 2016 and 2020 in five tertiary care hospitals across five cities in Saudi Arabia. *E. coli* (n=2190, 38.03%), *K. pneumoniae* (n=2154, 37.41%), *P. aeruginosa* (n = 918, 15.94%), and *A. baumannii* (n=496, 8.61%) constitute the 5758 gram-negative bacteria isolates. *E. coli* was the most frequently identified species in Riyadh, AlAhsa, Dammam, and Madinah (40%, 46.50%, 61.67%, and 43.66%, respectively), with a p-value of (p<0.001), except in Jeddah, where *K. pneumoniae* was the most prevalent (42%). The mean age of patients across Riyadh, AlAhsa, Dammam, and Madinah was 62.2 years (± 4.24). In contrast to Jeddah, where the majority of isolates (702; 41.8%) belonged to the adult age group. Most isolates were from male patients (3045; 52.9%), compared to 2713 (47.1%) from female patients. *K. pneumoniae* 1226 (40.3%) was the most prevalent isolate among male patients while *E. coli* (1135; 41.8%) was the most prevalent isolate among female patients.

Conclusion

Our study showed that the prevalence of carbapenem non-susceptible Gram-negative bacteria is relatively high, which therefore makes them very challenging to treat. The results show an urgent need for improved antibiotic stewardship strategies, including better surveillance and more effective infection control measures to reduce this issue. Further research into the molecular epidemiology and risk factors associated with these infections is necessary to guide public health policymakers in developing interventions to help control the spread of carbapenem-resistant Gram-negative bacteria.

## Introduction

Antibiotics significantly increased life expectancy and decreased mortality rates due to infections. However, this trend is starting to fade with the rise of multidrug-resistant organisms (MDR); these strains are becoming a global burden on healthcare and the economy [1,2]. The dramatic increase and spread of carbapenem-resistant gram-negative bacteria (CRGNB) has become a serious global public health concern [3].

The transition from susceptible pathogens to multiple drug resistance (MDR) pathogens has resulted in additional healthcare and economic burdens and will cost the world approximately $100 trillion over the next 30 years [2,4]. Antimicrobial resistance has increased at an alarming rate, as it has been estimated that MDR-related mortality will increase to 10 million by 2050 [3]. Resistance to third-generation cephalosporins, fluoroquinolones, and aminoglycosides has increased due to the spread of antimicrobial-resistant Gram-negative bacteria. In particular, the dramatic increase and spread of carbapenem-resistant-Gram-negative bacteria (CRGNB) has become a serious global public health concern, including in Saudi Arabia [5-8]. The World Health Organization (WHO) has classified CRGNB as a critical pathogen, indicating that it poses significant threats to human health and necessitates immediate research and development of novel antimicrobials [9-11].

Generally, bacteria have developed different mechanisms in which they become resistant, including enzymatic inactivations, mutations in the target site, and efflux pumps. The development of carbapenem resistance can be due to either one of two mechanisms; acquired or intrinsic resistance, and in some instances, both mechanisms can occur simultaneously. The enzymatic inactivations (acquired carbapenemases) are the most common mechanism for carbapenem resistance. This resistance mechanism includes (1) the destruction of carbapenems (which are resistant to hydrolysis by plasmid AmpCs) in conjunction with ESBL enzymes, (2) ESBL genes being transferred between organisms, and (3) mutation of porin with expression modulation. On the other hand, intrinsic resistance occurs by reducing uptake (due to modified porin channels) and by reducing the permeability of the membrane for B-lactam drugs. *Pseudomonas (P.) aeruginosa* organisms have a certain mechanism of resistance in which efflux pump overactivation is capitalized to develop resistance toward carbapenems [12]. The aim of the current study was, therefore, to estimate the rate of CRGNB-causing bacteremia in five tertiary hospitals in Saudi Arabia over the duration from 2016 to 2020.

## Materials and methods

Study setting, design, and subjects

The current study was conducted at King Abdulaziz Medical City (KAMC) and at King Abdullah International Medical Research Centre (KAIMRC), which are part of the Ministry of National Guard Health Affairs (MNGHA) in Saudi Arabia. MNGHA hospitals are tertiary healthcare hospitals that provide healthcare services to National Guard personnel and their families. Non-probability convenience sampling and a retrospective cross-sectional study were implemented, and all accessible medical records of patients with bacteremia due to carbapenem-resistant Gram-negative bacteria at five hospitals that met inclusion criteria between January 2016 and December 2020 were collected. The inclusion criteria consisted of patients of different age groups with confirmed diagnoses of bacteremia due to positive blood culture carbapenem-resistant Gram-negative bacteria (Escherichia (E.) coli, Klebsiella (K.) pneumonia, P. aeruginosa, and *Acinetobacter (A.) baumannii*). Data were gathered from the patient’s medical records, including demographics and microbiology laboratory findings. The total number of bacterial isolates in each age group and the rate of carbapenem resistance were calculated for 5,759 patients with CRGNB-related bacteremia.

Criteria for bacteremia diagnosis

A retrospective cross-sectional study was conducted in five tertiary care hospitals in Saudi Arabia among patients with bacteremia due to CRGNB. Electronic medical records (BestCare) were used to retrieve data regarding patient demographics and antimicrobial susceptibility testing (AST) over five years between January 2016 and December 2020. Patients with positive blood cultures for carbapenem-resistant *E. coli, K. pneumonia, P. aeruginosa*, and *A. baumannii* comprise the final study population.

Ethical considerations

The procedures followed in the current study were in accordance with the Declaration of Helsinki of 1975 (revised in 2000) and with the ethical standards of the institutional review board of KAIMRC in Riyadh, Saudi Arabia, where the study was conducted (protocol approval number: 1104/19, 7 July 2019). Permission was granted to access the patient’s medical records. As the current study was retrospective in nature, patient consent was waived.

Statistical analysis

Quantitative variables were presented as means with standard deviations while qualitative variables were presented as frequencies. The variables of the study were compared using the chi-square test. The information was analyzed using Microsoft Excel (2017) (Microsoft Corporation, Redmond, WA).

## Results

Bacterial isolates and demographics

**Figure 1:**
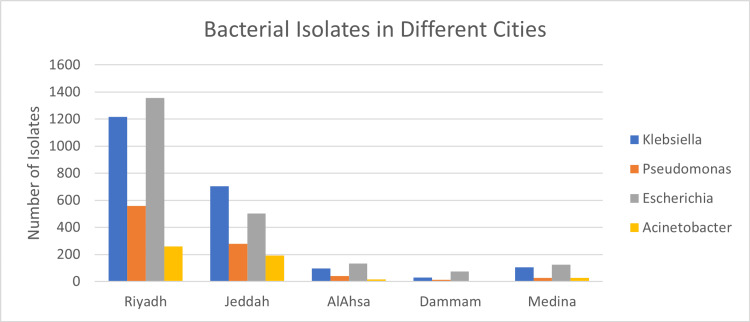
Illustration of the four gram-negative bacteria (K. pneumoniae, P. aeruginosa, A. baumannii, and E. coli) across cities E.: Escherichia; K.: Klebsiella; P.: Pseudomonas; A.: Acinetobacter

**Figure 2:**
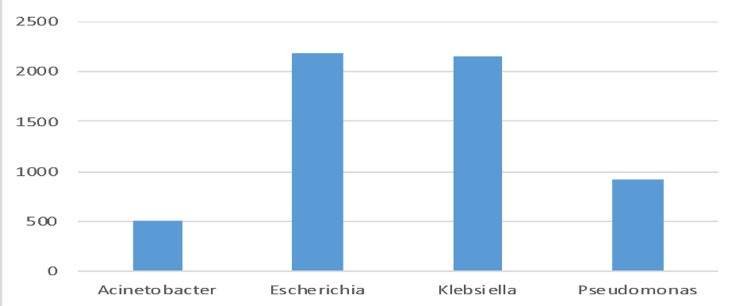
Illustrates the total number of isolates in blood cultures among the four species (n=5759)

**Figure 3:**
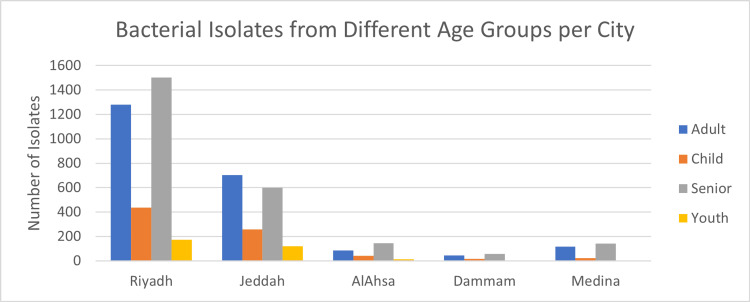
Demonstrates distribution of CR gram-negative bacteremia among different age groups according to each city CR: carbapenem resistance

**Figure 4:**
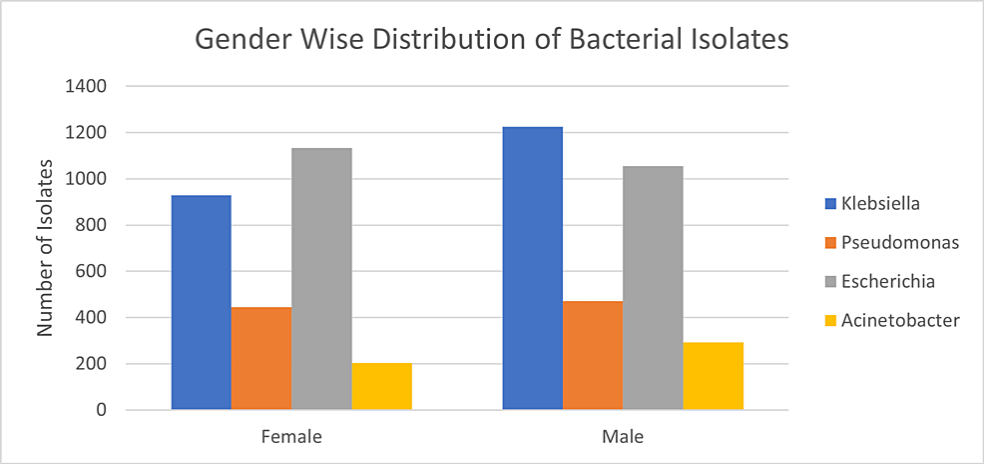
Illustrates CR gram-negative bacteremia among the four species according to gender CR: carbapenem resistance

This retrospective multi-centric study was conducted between 2016 and 2020 in five tertiary care hospitals across five cities in Saudi Arabia. Riyadh (n=3390, 58.86%), Jeddah (n=1678, 29.13%), AlAhsa, (n=286, 4.96%), Madinah (n=284, 4.93%), and Dammam (n=120, 2.08%) were among the regions from which the data were recovered (Table 1 and Figure 1). *E. coli* (n=2190, 38.03%), *K. pneumoniae* (n=2154, 37.41%), *P. aeruginosa* (n=918, 15.94%), and *A. baumannii* (n=496, 8.61%) constitute the 5758 gram-negative bacteria isolates (Figure 2). *E. coli *was the most frequently identified species in Riyadh, AlAhsa, Dammam, and Madinah (40%, 46.50%, 61.67%, and 43.66%, respectively), except in Jeddah, where *K. pneumoniae* was the most prevalent (42%). The mean age of patients across Riyadh, AlAhsa, Dammam, and Madinah was 62.2 years (± 4.24), with 42.4% of patients being elderly (age over 65), 38.7% being adults (age between 25 and 64), 5.5% being youth (age between 15 and 24), and 13.4% being children (age below 15). Riyadh had 1,503 (44.3%), AlAhsa 145 (50.7%), Dammam 56 (46.7%), and Madinah 141 (49.6%) older adults among the total number of isolated individuals (Table 2). In contrast to Jeddah, where the majority of isolates, 702 (41.8%), belonged to the adult age group illustrated in Figure 3 (P < 0.001). Most isolates were from male patients, 3045 (52.9%), compared to 2713 (47.1%) from female patients (Table 2). *K. pneumoniae* 1226 (40.3%) was the most prevalent isolate among male patients, while *E. coli* 1135 (41.8%) was the most prevalent isolate among female patients, as shown in Figure 4 (P < 0.001).

**Table 1:** Distribution of positive blood culture among five cities and frequency of each bacteria in each city. E.: Escherichia; K.: Klebsiella; P.: Pseudomonas; A.: Acinetobacter

Species	City					
	Riyadh (n)	Jeddah (n)	AlAhsa (n)	Dammam (n)	Medina (n)	Total (n)
K. pneumoniae	1217	705	96	30	106	2154
E. coli	1356	503	133	74	124	2190
P. aeruginosa	559	278	41	14	26	918
A. baumannii	258	192	16	2	28	496
Total	3390	1678	286	120	284	5758

**Table 2:** Shows the distribution of patient age and gender across the five cities

Age in Years	Cross all cities (Mean ± SD)	Elderly age over 65 (%)	Adult ages 25-64 (%)	Youth ages 15-24 (%)
	62.2±4.24	42.4	38.7	5.5
Gender		n (%)		
Male		3045 (52.9)		
Female		2713 (47.1)		
Cities		n (%)		
Riyadh		3390 (58.86)		
Jeddah		1678 (29.13)		
Madinah		284 (4.93)		
Dammam		120 (2.08)		
AlAhsa		1678 (29.13)		

Carbapenem resistance (CR) rate

**Figure 5:**
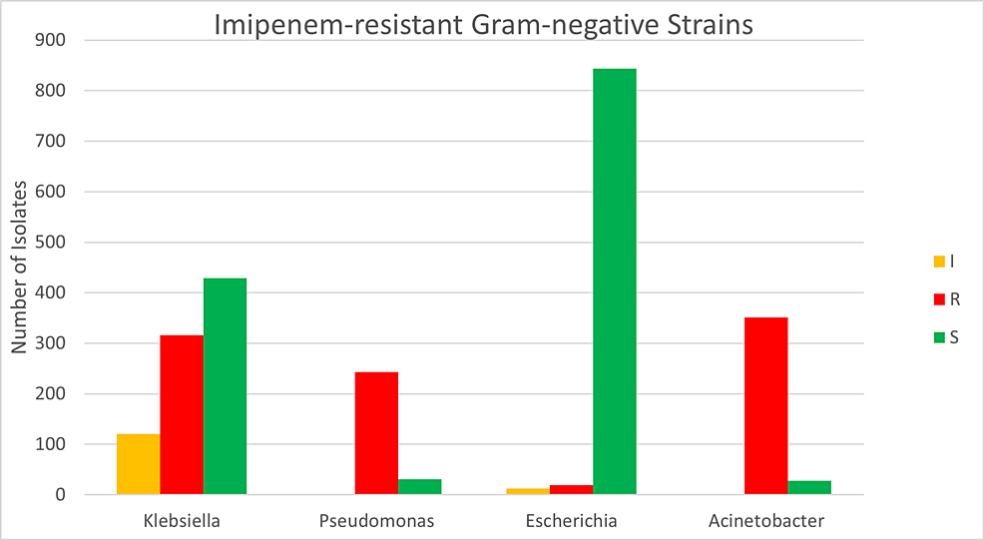
Illustrates degrees of resistance to imipenem among the four species Intermediate (I), Resistant (R), Sensitive (S)

**Figure 6:**
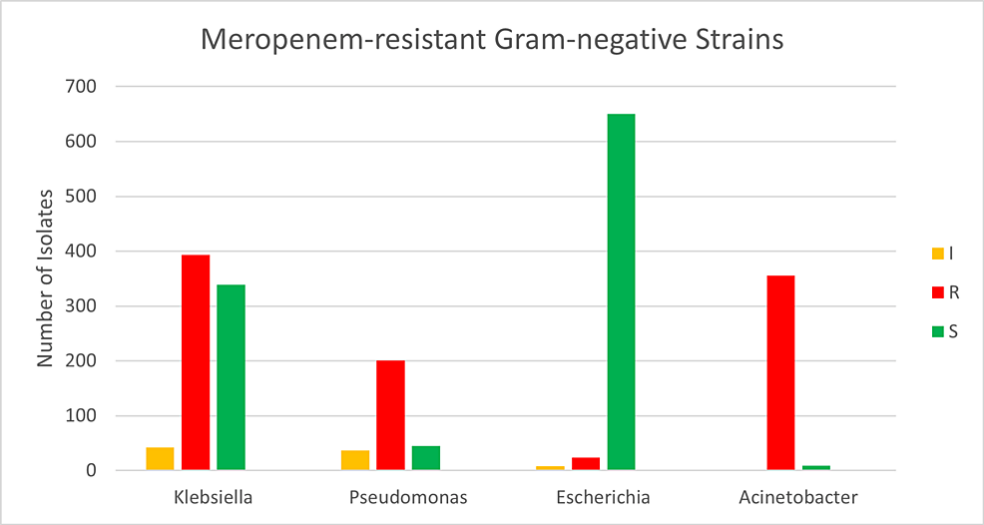
Illustrates degrees of resistance to meropenem among the four species Intermediate (I), Resistant (R), Sensitive (S)

Overall, the CR rate for the four pathogens was 38% (929/2396) for imipenem and 46% (973/2104) for meropenem. *A. baumannii* illustrates the highest level of resistance to Imipenem; out of 381 samples, 351 (92%) were resistant, whereas 243 (88%) of 275 *P. aeruginosa* samples were resistant. Among 865 samples of *K. pneumoniae*, 316 (37%) were resistant, and 120 (14%) exhibited intermediate resistance to imipenem. *E. coli* had the highest number of tested samples (n=875), of which 844 (96%) were susceptible to imipenem (P<0.001) (Table 3 and Figure 5). Resistance to meropenem exhibits a similar pattern, as shown in Table 4 and Figure 6.

**Table 3:** Illustrates degrees of resistance to meropenem among the four species Intermediate (I), Resistant (R), Sensitive (S) E.: Escherichia; K.: Klebsiella; P.: Pseudomonas; A.: Acinetobacter

Meropenem	I	R	S	Total
K. pneumoniae	42	393	339	774
E. coli	8	24	650	682
P. aeruginosa	37	201	45	283
A. baumannii	1	355	9	365
Total	88	973	1043	2104

**Table 4:** Illustrates degrees of resistance to imipenem among the four species Intermediate (I), Resistant (R), Sensitive (S) E.: Escherichia; K.: Klebsiella; P.: Pseudomonas; A.: Acinetobacter

Imipenem	I	R	S	Grand Total
K. pneumoniae	120	316	429	865
E. coli	12	19	844	875
P. aeruginosa	1	243	31	275
A. baumannii	2	351	28	381
Total	135	929	1332	2396

## Discussion

Our research revealed that *E. coli* was the most prevalent organism discovered in blood cultures. However, a different study conducted by Ayobami (2019) at MNGHA between 2006 and 2017 contradicts this result; the study showed that *P. Aeruginosa* was the most prevalent organism in blood cultures taken between 2006 to 2017 [9]. Moreover, our research demonstrates that the incidence of *E. coli* infections has risen. Similarly, a study conducted by Miranda (2020) in Mexico between 2016 and 2017 on 4382 blood isolates revealed that *E. coli* and *K. pneumoniae* are the most prevalent gram-negative species, exhibiting over 30% resistance to most commonly used antibiotics and less than 20% resistance to carbapenem [13].

The mean age of patients was 62.2, with seniors comprising 42.4% of the patient population, followed by adults comprising 38.8%. This may result from an increase in co-morbidities, hospitalizations, and interventions. Most of the isolates were obtained from male patients (52.9%), which was consistent across all Gram-negative bacteria, except for *E. coli*, where female isolates were more prevalent. The prevalence of urinary tract infections among females may have contributed to these results. Comparatively, males had a higher prevalence of gram-negative bacteria worldwide [2]. Except for *E. coli*, all isolates exhibited high resistance to carbapenem. This is somewhat concerning and may be related to the increased use of antibiotics over the past few decades. In 2010, a study conducted in a hospital in Riyadh, Saudi Arabia, revealed an overuse of antimicrobial agents in four adult intensive care units (ICUs), with meropenem being the most commonly used antibiotic (33.2 defined daily doses (DDD) per 100 bed-days) [14].

A cross-sectional study conducted by Yang (2018) in China across 153 hospitals linked CRGNB and antibiotic consumption intensity, finding that increased use of meropenem has been related to the rate of carbapenem-resistant *K. pneumonia* (CRKP). Carbapenemases such as New Delhi Metallo-lactamase-1 (NDM-1) And *K. pneumoniae* carbapenemases (KPC) are produced by increased carbapenem usage; the study linked Carbapenem resistance to increased antibiotic consumption in all remaining species [15].

In Europe, a 2017 study surveyed carbapenem resistance (CR) across three species: *K. pneumonia*, *P. aeruginosa*, and *A. baumannii*. The study revealed a disparity in resistance profiles between Western and Eastern Europe, which may be attributable to antibiotic use. Globally, CR for *A. baumannii* is typically quite high, whereas some European nations have lower resistance. It ranges from 0% in Belgium to 95% in Greece. CR rates are typically collected from a single region or hospital, so additional research is required [16]. This also emphasizes the significance of global AMR surveillance programs. Fortunately, new policies and regulations have addressed this issue head-on in recent years. Nationwide antimicrobial stewardship and surveillance programs are utilized to combat this problem.

A systemic review and meta-analysis in 2017 revealed a correlation between CR and *K. pneumonia* mortality rate. There were 2,167 patients across 15 studies, of which 1,148 had CSKP bacteremia and 1,019 had CRKP bacteremia. CRKP bacteremia patients had a higher mortality rate than CSKP bacteremia patients [17].

## Conclusions

Our study showed that the prevalence of carbapenem non-susceptible Gram-negative bacteria is relatively high, which therefore makes them very challenging to treat. The results show an urgent need for improved antibiotic stewardship strategies, including better surveillance and more effective infection control measures to reduce this issue. Antimicrobial resistance (AMR) trends must be identified on a national and international scale, necessitating routine surveillance studies to provide crucial data. Further research into the molecular epidemiology and risk factors associated with these infections is necessary to guide public health policymakers in developing interventions to help control the spread of carbapenem-resistant Gram-negative bacteria. Further research is recommended to establish a connection between CR and clinical outcomes and mortality rate.
